# An Unusual Case of Cutaneous Ewing Sarcoma of the Anterior Abdominal Wall in an Adult Patient

**DOI:** 10.7759/cureus.78848

**Published:** 2025-02-11

**Authors:** Mena Louis, Nicholas Minner, Evan Weitman

**Affiliations:** 1 General Surgery, Northeast Georgia Medical Center Gainesville, Gainesville, USA; 2 Surgery, Edward Via College of Osteopathic Medicine, Spartanburg, USA; 3 Surgery, Piedmont Atlanta, Atlanta, USA

**Keywords:** abdominal wall mass, cd56, cutaneous ewing sarcoma, ewsr1-fli1 fusion gene, gata3, small round blue cell tumor

## Abstract

Ewing sarcoma is a malignant small round blue cell tumor most commonly found in the bones of children and adolescents. Cutaneous Ewing sarcoma, originating in the skin and subcutaneous tissues, is exceedingly rare, especially in adults. We present the case of a 57-year-old woman who developed a progressively enlarging mass on her anterior abdominal wall. The lesion grew over several months and became increasingly erythematous. The mass was initially misdiagnosed as a soft tissue infection and the patient was treated with antibiotics followed by attempted incision and drainage with no clinical resolution. Imaging studies, including a contrast-enhanced CT scan, revealed a lobulated heterogeneous soft tissue mass measuring 5 x 3.9 x 5.2 cm, abutting and possibly infiltrating the left rectus muscle.

The patient subsequently underwent radical resection with close but negative margins. Histopathological examination demonstrated small round blue cells arranged in solid sheets with associated vasculature. Immunohistochemical staining was positive for GATA3 and showed CD56 positivity, while negative for multiple other markers, aiding in excluding alternative diagnoses. Molecular studies confirmed the diagnosis of Ewing sarcoma. Postoperative management involved a multidisciplinary approach, including plans for systemic chemotherapy and consideration of adjuvant radiation therapy due to the high risk of local recurrence in the setting of close margins.

Cutaneous Ewing sarcoma typically presents as a rapidly enlarging mass that may resemble benign conditions such as sebaceous cysts or inflammatory processes. The key to its identification is histopathological evaluation, including immunohistochemical staining, as well as molecular testing to detect characteristic genetic translocations like the EWSR1-FLI1 fusion gene. Early and accurate diagnosis is crucial due to the tumor's aggressive nature and high potential for metastasis. Implementing a multidisciplinary treatment plan encompassing surgery, chemotherapy, and possibly radiation therapy is essential for improving patient outcomes.

## Introduction

Ewing sarcoma is a malignant tumor that primarily affects children and young adults, originating most commonly in the bones [1]. However, it can also arise in soft tissues, known as extraskeletal Ewing sarcoma, which includes rare occurrences in the skin and subcutaneous tissues [2]. Cutaneous presentations are uncommon and can be easily overlooked or misdiagnosed due to their rarity and nonspecific clinical features [3].

Recognizing cutaneous Ewing sarcoma is vital because its clinical appearance often resembles benign skin lesions or other malignancies [4]. It typically presents as a small, firm mass that may grow rapidly, sometimes accompanied by redness or tenderness [5]. Due to these nonspecific symptoms, there can be delays in diagnosis, which may adversely affect the patient's prognosis [6].

Advancements in diagnostic methods, including immunohistochemistry and molecular genetic testing, have improved the ability to accurately identify this rare tumor [7]. Understanding the characteristics of cutaneous Ewing sarcoma can aid clinicians in distinguishing it from other skin lesions, ultimately improving patient outcomes.

## Case presentation

A 57-year-old woman with a history of hyperlipidemia, fibromyalgia, depression, and anxiety presented with a small lesion on her anterior abdominal wall. Initially the size of a peanut, the lesion progressively enlarged over several months, becoming increasingly erythematous, which prompted her to seek medical attention. The patient reported no systemic symptoms preceding her clinical evaluation. She has no history of tobacco, alcohol, or illicit drug use, and denied any pertinent medical or family history of malignancies.

**Figure 1:**
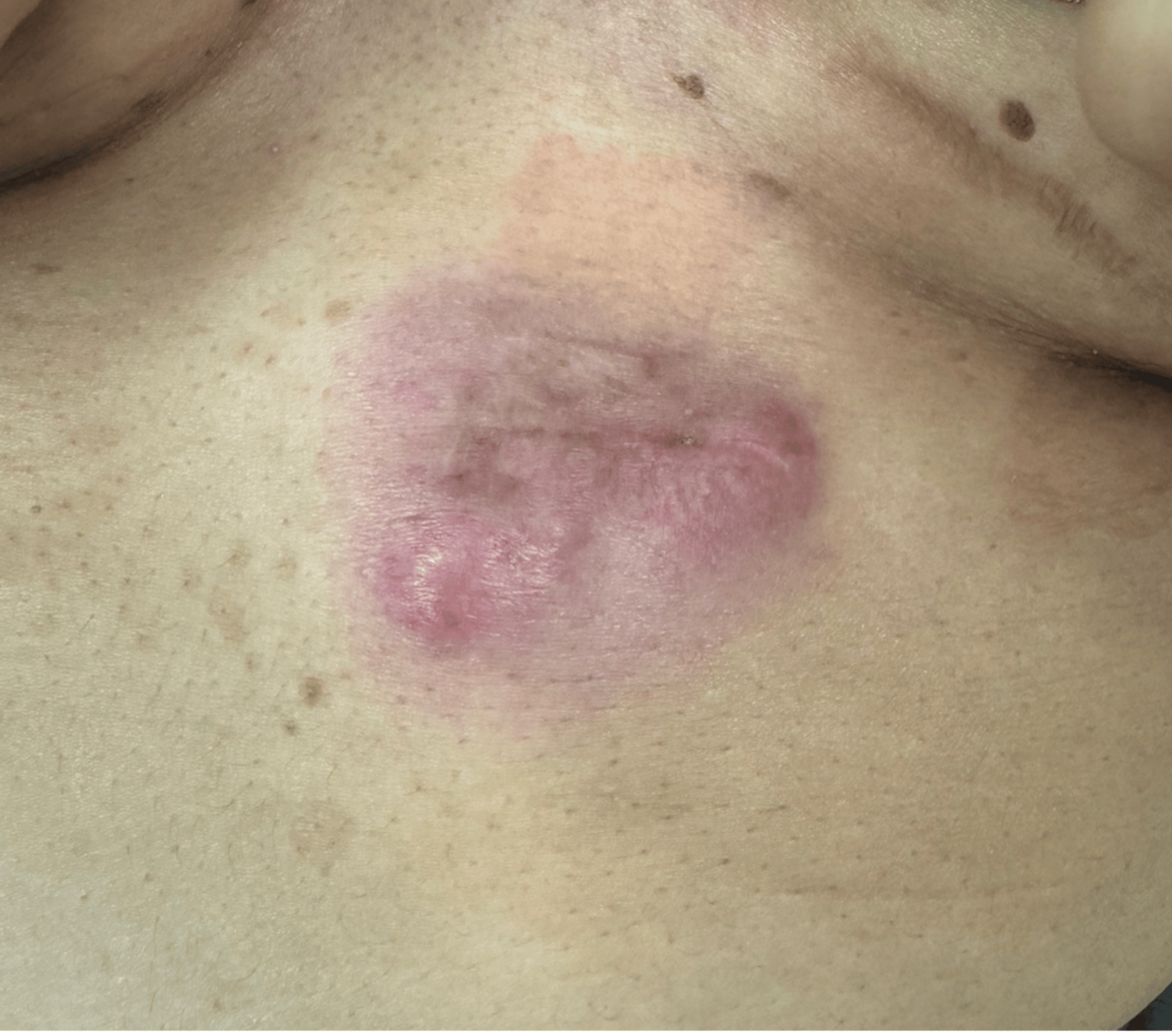
Anterior abdominal wall mass

Her primary care physician initially prescribed a course of cephalexin followed by incision and drainage, which was aborted due to the high vascularity of the mass. Physical examination revealed a lobulated, heterogeneous mass approximately 5 centimeters in size on the left side of her anterior abdominal wall (Figure 1). The overlying skin was red and swollen, but there were no signs of systemic infection or lymphadenopathy.

**Figure 2:**
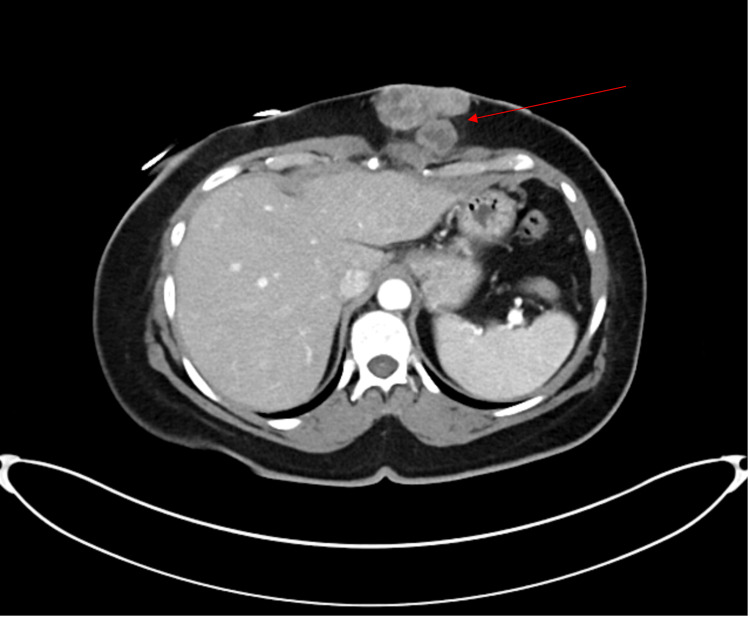
Axial contrast-enhanced CT scan of the abdomen and pelvis (CTAP) showing a 5.2 × 3.9 cm lobulated, heterogeneous soft tissue mass (red arrow) in the anterior abdominal wall, primarily within the subcutaneous fat. The mass abuts and possibly infiltrates the rectus muscle without deeper extension.

**Figure 3:**
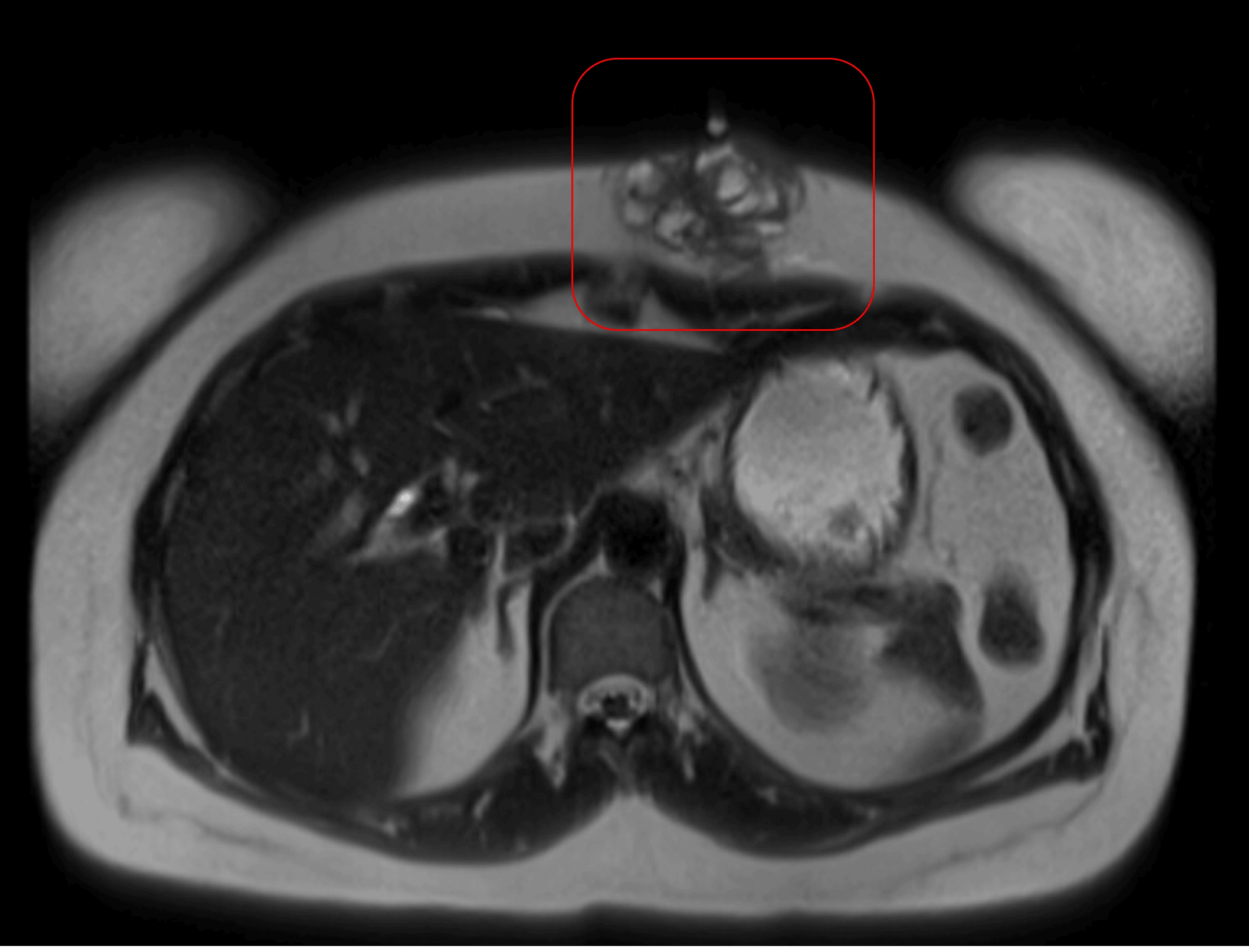
Axial T2-weighted MRI scan of the abdomen showing a subcutaneous enhancing mass (red square) in the left upper anterior abdominal wall.

Contrast-enhanced CT imaging of the abdomen and pelvis revealed a 5.2 x 3.9 x 5.0 cm lobulated, heterogeneous soft tissue mass within the left anterior abdominal wall that abutted and possibly infiltrated the left rectus muscle, without extending deeper (Figure 2). A contrast-enhanced MRI of the abdomen confirmed a subcutaneous enhancing mass in the same location (Figure 3).

**Figure 4:**
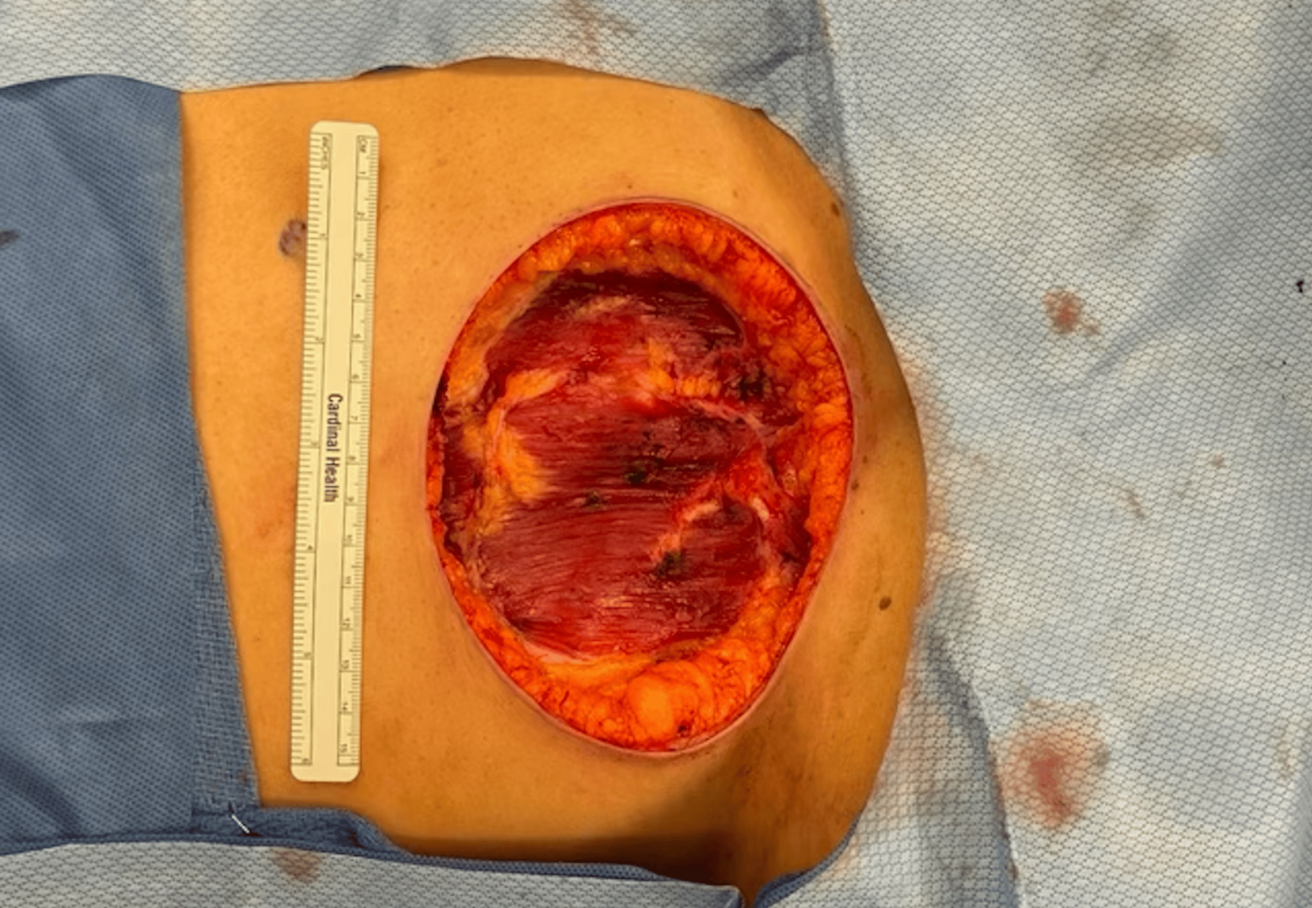
Intraoperative photograph showing the surgical site following the complete resection of the anterior abdominal wall mass.

Given the concerning imaging findings and the progressive enlargement of the mass, a thorough metastatic evaluation was performed - encompassing imaging of the chest and abdomen - to rule out any additional primary or metastatic lesions. No evidence of distant disease was identified. A core needle biopsy was conducted beforehand to confirm the histopathological diagnosis. Subsequently, the patient underwent a radical resection of the mass, including en bloc partial resection of the anterior abdominal wall. A one-centimeter margin was achieved circumferentially, although the final pathology revealed close but clear margins (0.5 cm at the deep aspect). The resulting defect measured 14 × 11 cm and was initially managed with negative-pressure wound therapy (Figure 4). Definitive closure was planned as a staged procedure using a biologic mesh graft after confirming negative margins and ensuring the absence of recurrent disease. Additional re-resection was not pursued, as the margins were deemed adequate for local control in the context of planned adjuvant therapy. The patient tolerated the procedure well and was discharged home the following morning.

Histopathological examination revealed small round blue cells arranged in solid sheets with associated vasculature. Immunohistochemical staining was positive for GATA3 and showed some CD56 positivity, while the tumor cells were negative for cytokeratin 7 and 20, chromogranin, synaptophysin, mammaglobin, estrogen receptor, P63, P40, CD45, Pax5, epithelial membrane antigen, desmin, smooth muscle actin, thyroid transcription factor-1, and Wilms tumor protein 1. Fluorescence in situ hybridization (FISH) for DDIT3/GLI1 (12q13.3) was within the reference range, ruling out myxoid liposarcoma. EWSR1 rearrangement testing was not available at our institution, but the overall histopathological and immunohistochemical findings strongly supported a diagnosis of Ewing sarcoma.

Before starting further therapy, the patient underwent a staging workup that included CT scans of the chest and abdomen, as well as relevant laboratory evaluations, all of which showed no metastatic disease. After the final pathology result confirmed Ewing sarcoma, a comprehensive, multidisciplinary treatment plan was initiated. Although standard regimens for Ewing sarcoma typically involve vincristine, doxorubicin, cyclophosphamide, ifosfamide, and etoposide (VDC/IE), the medical oncology team recommended a six-cycle CHOP regimen (cyclophosphamide, doxorubicin, vincristine, and prednisone) in light of the patient’s comorbidities and tolerance concerns. This approach was determined through collaboration among surgical, medical, and radiation oncology specialties to best address the patient’s clinical needs and treatment objectives.

## Discussion

Soft tissue masses of the abdominal wall present a diagnostic challenge due to the wide range of potential etiologies, from benign cysts to malignant tumors [8]. A systematic approach to evaluation is essential, beginning with a thorough physical examination [9]. Clinicians should assess the size, consistency, mobility, tenderness, and any overlying skin changes of the mass. In this case, the lesion was firm, rapidly enlarging, and associated with redness features that raise suspicion for a neoplastic process.

The presence of a firm, rapidly growing mass that does not respond to conventional therapies, such as antibiotics, warrants further investigation for malignancy [10]. Prior attempts at incision and drainage were aborted due to high vascularity, a finding that can indicate aggressive tumor biology [11]. High vascularity complicates surgical interventions and suggests the need for careful preoperative planning [12].

Imaging studies are crucial in characterizing abdominal wall masses and guiding management [13]. Ultrasound may serve as an initial modality but has limitations in depth and resolution [14]. CT scans provide detailed information about the lesion's size, location, and involvement of adjacent structures [15]. In this patient, contrast-enhanced CT imaging revealed a heterogeneous mass abutting and possibly infiltrating the anterior abdominal wall muscles.

Magnetic resonance imaging (MRI) offers superior soft tissue contrast and is invaluable in characterizing soft tissue tumors, including sarcomas. Contrast-enhanced MRI can assess the lesion's vascularity, delineate its margins, and evaluate the involvement of surrounding tissues. MRI findings such as heterogeneity, infiltration into adjacent structures, and high vascularity suggest malignancy. The specific value of MRI lies in its ability to aid in diagnosis and surgical planning [16].

A core needle biopsy is essential for obtaining tissue for definitive diagnosis. In this case, the biopsy revealed small round blue cells arranged in solid sheets with associated vasculature. Immunohistochemical staining was positive for GATA3 and CD56 and negative for a broad panel of other markers. Molecular studies further supported the diagnosis of Ewing sarcoma.

The differential diagnosis for soft tissue masses of the abdominal wall is broad. It includes benign lesions such as lipomas, epidermoid cysts, abscesses, and desmoid tumors, as well as malignant entities like soft tissue sarcomas, lymphomas, and metastatic carcinomas [17]. High vascularity and rapid growth are features more commonly associated with malignancies [12]. It is also crucial to determine whether the mass is a primary tumor or a metastatic focus of the disease. Metastases to the abdominal wall can originate from intra-abdominal malignancies or distant sites [18].

Adopting the principle of “Name it, stage it, treat it” involves a multidisciplinary effort that includes surgical, medical, and radiation oncology teams working together to determine the most appropriate diagnostic and therapeutic strategies [19]. This approach begins with imaging and biopsy to identify the nature of the mass, followed by thorough staging to assess local invasion and possible distant disease using modalities such as CT and MRI. In this case, a PET/CT scan was not performed because the CT and MRI findings, combined with a core needle biopsy, provided sufficient information to establish the diagnosis and guide the treatment plan. Accurate staging through coordinated evaluation informs both the treatment approach and prognosis, ensuring that each specialty’s expertise contributes to optimal patient care.

Clinicians should suspect malignancy when an abdominal wall mass demonstrates certain concerning characteristics. Firmness on examination may suggest dense cellularity, indicating a possible malignant process. A rapidly enlarging lesion that grows noticeably over weeks to months raises additional concern. Imaging findings such as irregular borders, necrotic areas, and variable enhancement patterns further support the possibility of a malignant tumor. Increased vascularity often reflects aggressive behavior and should not be overlooked. Finally, failure to respond to initial treatments, such as antibiotics, or recurrence after a drainage procedure, provides yet another clue that the lesion may be malignant rather than benign.

While Ewing sarcoma predominantly arises in the bones of children and adolescents - known as bony or osseous Ewing sarcoma - it can also occur in soft tissues, including the skin [20]. Bony Ewing sarcoma typically presents with localized bone pain and swelling, often affecting the long bones of the extremities, pelvis, or ribs [1]. Systemic symptoms such as fever, weight loss, and anemia may accompany the primary symptoms due to the aggressive nature of the tumor [21]. Radiographically, bony lesions often exhibit characteristic features like a permeative pattern of bone destruction and a layered periosteal reaction, sometimes described as an "onion-skin" appearance [5].

In contrast, cutaneous Ewing sarcoma is exceedingly rare and can present across a broader age range, including adults, as illustrated in this case [20]. Cutaneous lesions manifest as superficial masses in the skin or subcutaneous tissue, often without initial systemic symptoms. They may be mistaken for benign conditions like cysts or infections, leading to delays in diagnosis. Despite the differences in typical age of onset and primary location, both bony and cutaneous Ewing sarcomas share similar histopathological features, including small round blue cells that are positive for CD99 on immunohistochemistry [22].

In diagnosing Ewing sarcoma, immunohistochemical studies are commonly utilized to help distinguish it from other small round blue cell tumors, each of which has a unique profile. For example, lymphoma is typically positive for leukocyte common antigen (CD45), whereas rhabdomyosarcoma expresses desmin and myogenin. Neuroblastoma often shows synaptophysin and chromogranin, while desmoplastic small round cell tumors co-express cytokeratins and desmin. Merkel cell carcinoma features cytokeratin 20 with a characteristic perinuclear dot pattern. In contrast, Ewing sarcoma generally demonstrates strong membranous positivity for CD99 and may also show FLI-1, assisting in differentiation from other entities.

Ewing sarcoma typically shows strong membranous positivity for CD99 (MIC2) and may express FLI-1, aiding in its identification [23]. The tumor cells in this case exhibited strong membranous positivity for CD99 (MIC2). However, CD99 is not entirely specific and can be expressed in other tumors. Additional markers such as FLI-1 and NKX2.2 can aid in diagnosis. The expression of GATA3 in this case is atypical but has been documented in some instances of Ewing sarcoma [24]. The negative staining for cytokeratins, desmin, myogenin, and leukocyte common antigen helps exclude carcinoma, rhabdomyosarcoma, and lymphoid malignancies, respectively.

Molecular genetic testing is definitive for diagnosing Ewing sarcoma. The characteristic translocation t(11;22)(q24;q12) results in the EWSR1-FLI1 fusion gene, present in approximately 85% of cases [25]. Detection of this fusion gene through techniques like FISH or reverse transcription-polymerase chain reaction (RT-PCR) confirms the diagnosis and can have prognostic significance [26]. In this case, the FISH study for DDIT3/GLI1 was within the reference range, which is more relevant for excluding myxoid liposarcoma.

Neoadjuvant chemotherapy is frequently considered to reduce tumor size before surgery, especially when there is concern about achieving adequate margins or the tumor’s anatomic location complicates resection. In this case, the lesion was deemed resectable with a reasonable likelihood of obtaining clear margins, and urgent surgical management was prioritized to prevent further progression. Once the pathology confirmed Ewing sarcoma, the patient was transitioned to systemic chemotherapy to address any microscopic metastatic disease. Surgical resection with clear margins is essential, as performed in this case. Given the high risk of local recurrence and distant metastatic disease, systemic chemotherapy is a mainstay of treatment [27,28].

Surgical resection with clear margins is fundamental in managing Ewing sarcoma, given its high risk of local recurrence and distant metastasis. Although standard chemotherapy regimens typically incorporate VDC/IE, in this case, the patient was prescribed a six-cycle CHOP regimen. CHOP is more commonly associated with non-Hodgkin lymphoma but was selected here due to specific patient-related factors, including comorbidities and treatment tolerance concerns that made VDC/IE less suitable. This plan was finalized through a multidisciplinary discussion involving surgical, medical, and radiation oncology teams. Radiotherapy was also considered to further enhance local control, particularly given the tumor’s size and the close surgical margins.

However, the prognosis for cutaneous Ewing sarcoma may be more favorable due to earlier detection and the possibility of achieving clear surgical margins [28]. Cutaneous lesions are more accessible and may be detected before significant metastasis occurs. In contrast, bony Ewing sarcoma often presents at a more advanced stage due to deeper location and non-specific symptoms [29].

Despite these differences, long-term outcomes for cutaneous Ewing sarcoma still require careful monitoring due to the risk of metastasis. For localized bony Ewing sarcoma, five-year survival rates have reached 70-80%, but the prognosis is less favorable in older adults and those with metastatic disease at diagnosis [28]. Cutaneous Ewing sarcoma's rarity means data are limited, but some studies suggest a better prognosis compared to osseous forms.

## Conclusions

Cutaneous Ewing sarcoma is an uncommon form of a tumor more frequently seen in bone, and it requires an attentive diagnostic process, particularly when skin lesions resist usual treatments. Swift assessment through thorough examination, advanced imaging, and biopsy is key to identifying this malignancy at an earlier stage. A multidisciplinary strategy involving complete surgical removal with adequate margins, chemotherapy, and possibly radiation therapy remains the cornerstone of effective management. Some reports suggest that cutaneous presentations may have a better outcome compared to osseous forms, potentially due to earlier detection and more feasible surgical resection. However, data on cutaneous Ewing sarcoma are limited, and caution is advised when interpreting individual cases. Ongoing research and expanded case reporting are needed to clarify long-term prognosis and refine therapeutic recommendations.
